# Naturopathy, complementary and integrative medicine in medical education – position paper by the GMA Committee Integrative Medicine and Perspective Pluralism

**DOI:** 10.3205/zma001537

**Published:** 2022-04-14

**Authors:** Angelika Homberg, Christian Scheffer, Benno Brinkhaus, Ulrike Fröhlich, Roman Huber, Stefanie Joos, Petra Klose, Klaus Kramer, Miriam Ortiz, Matthias Rostock, Jan Valentini, Beate Stock-Schröer

**Affiliations:** 1 Medical Faculty Mannheim of Heidelberg University, Department for Medical Education Research, Mannheim, Germany; 2 University of Witten/Herdecke, Integrated Curriculum of Anthroposophic Medicine, Witten, Germany; 3 Charité - University Medicine Berlin, corporate member of Freie Universität Berlin and Humboldt University of Berlin, Institute for Social Medicine, Epidemiology and Health Economics, Berlin, Germany; 4 Hahnemann Association of Homeopathic Physicians, Wiesbaden, Germany; 5 University Hospital Freiburg, University Center for Naturopathy, Freiburg, Germany; 6 University Hospital Tübingen, Institute for General Medicine and Interprofessional Care, Tübingen, Germany; 7 Evang. Kliniken Essen-Mitte, Clinic for Naturopathy and Integrative Medicine, Essen, Germany; 8 University Hospital Ulm, Department of Integrative Medicine, Clinic for General and Visceral Surgery, Ulm, Germany; 9 University Hospital Hamburg-Eppendorf, University Cancer Center Hamburg, Hubertus Wald Tumor Center, Hamburg, Germany

**Keywords:** medical training, curriculum development, skills, naturopathy, complementary medicine, integrative medicine, evidence-based medicine, health, patient orientation, interprofessional education

## Abstract

**Background:**

A large part of the population in Germany makes use of naturopathic, complementary and integrative medical treatments. There are now numerous scientific studies that provide evidence of efficacy for certain indications. At German medical faculties, selected procedures and their application are taught within the cross-sectoral unit called QB 12 and some elective courses, with a focus on specific aspects are offered. So far, however, there has been no structured curriculum that longitudinally anchors teaching across medical studies and enables all students to consider naturopathic and complementary medical options for patient care later on and to integrate them effectively into the diagnostic and treatment process.

**Objective:**

The aim of this position paper is to show the relevance of this topic for medical education, to clarify terminology and to present core competencies and possible implementation options for training.

**Method:**

The Integrative Medicine and Perspective Pluralism Committee of the German Association for Medical Education developed this position paper in a multi-stage consensual process, in cooperation with the Forum of University Work Groups on Naturopathic Treatment and Complementary Medicine.

**Results:**

First, different umbrella terms were discussed and an existing definition of integrative medicine and health was chosen for subsequent use. Building on this step, the status of education and its scientific foundation in Germany was considered in an international context. In the next step, a competency profile for medical training, consisting of seven areas of competency, was developed and described in detail with regard to naturopathic, complementary and integrative medicine. Implementation options were identified using possible starting points in the curriculum and using established examples of best practice.

**Conclusion:**

Despite different priorities at each faculty, it was possible to find an agreement on the development of competencies and anchoring them in medical education on the basis of a common definition of terms. Currently, the implementation in the mandatory and elective areas is very heterogeneous. As part of the current revision of the Medical Licensure Act, there are many possible starting points for the integration of naturopathic and complementary medical teaching content, especially in interprofessional and general practice courses. The implementation and accompanying research of targeted teaching settings should lay the foundations for a long-term and binding integration into medical education. Overall, it is clear that medical education in the field of naturopathy and complementary and integrative medicine has the potential to develop comprehensive core medical competencies.

## Zusammenfassung

**Hintergrund:**

Ein großer Anteil der Bevölkerung in Deutschland nimmt naturheilkundliche, komplementär- und integrativmedizinische Therapieverfahren in Anspruch. Mittlerweile existieren zahlreiche wissenschaftliche Studien, die einen Wirksamkeitsnachweis bei bestimmten Indikationen liefern. An deutschen Medizinischen Fakultäten werden ausgewählte Verfahren und deren Anwendung im Rahmen des Querschnittsbereichs 12 gelehrt, an einigen werden Wahlfächer mit eigenen Schwerpunktsetzungen angeboten. Bislang fehlt jedoch ein strukturiertes Curriculum, welches longitudinal die Lehre über das Medizinstudium hinweg verankert und alle Studierende befähigt, naturheilkundliche und komplementärmedizinische Optionen in der späteren Patientenversorgung zu berücksichtigen und wirksam in den Diagnostik- und Therapieprozess einzubinden.

**Zielsetzung:**

Ziel dieses Positionspapiers ist, die Relevanz des Themas für die medizinische Ausbildung darzustellen, begriffliche Klarheit zu schaffen sowie Kernkompetenzen und mögliche Implementierungsmöglichkeiten für die Ausbildung vorzustellen.

**Methode:**

Der Ausschuss Integrative Medizin und Perspektivenpluralismus der Gesellschaft für Medizinische Ausbildung erarbeitete in Zusammenarbeit mit dem Forum universitärer Arbeitsgruppen Naturheilverfahren und Komplementärmedizin das vorliegende Positionspapier in einem mehrstufigen konsensualen Prozess.

**Ergebnisse:**

Zunächst wurden unterschiedliche Oberbegriffe diskutiert und für die weitere Arbeit eine bestehende Definition für Integrative Medizin und Gesundheit ausgewählt. Darauf aufbauend wurde der Stand der Ausbildung und deren wissenschaftliche Fundierung in Deutschland im internationalen Kontext reflektiert. In einem weiteren Schritt wurde ein Kompetenzprofil für die medizinische Ausbildung, bestehend aus sieben Kompetenzbereichen, entwickelt und im Hinblick auf Naturheilverfahren, komplementäre und integrative Medizin detailliert dargestellt. Implementierungsmöglichkeiten wurden anhand möglicher Anknüpfungspunkte im Curriculum sowie anhand bereits etablierter Best-Practice-Beispiele aufgezeigt.

**Schlussfolgerung:**

Trotz unterschiedlicher Schwerpunktsetzungen an den einzelnen Fakultäten ist es gelungen, auf Grundlage einer gemeinsamen Begriffsdefinition eine Übereinkunft über die mögliche Kompetenzentwicklung und Verankerung in der medizinischen Ausbildung zu finden. Aktuell ist die Umsetzung in den Pflicht- und Wahlbereichen sehr heterogen. Im Rahmen der aktuellen Überarbeitung der Approbationsordnung bieten sich vielfache Anknüpfungspunkte für eine Einbindung naturheilkundlicher und komplementärmedizinischer Lehrinhalte insbesondere bei interprofessionellen und allgemeinmedizinischen Lehrangeboten. Durch die Implementierung und Begleitforschung zielgerichteter Lehr-Settings sollten die Grundlagen für eine langfristige und verbindliche Integration in die medizinische Ausbildung gelegt werden. Insgesamt wird deutlich, dass die Lehre im Bereich Naturheilverfahren und komplementäre und integrative Medizin das Potential zur Entwicklung umfangreicher ärztlicher Kernkompetenzen hat.

## Preface

The World Health Organization (WHO) highlighted the important role of traditional (naturopathic) medicine in health care in 1978 [1] and continues to recognize it as culturally acceptable, affordable and sustainable medicine [2]. Nevertheless, corresponding care concepts have been largely neglected in large-scale international health programs [3], [4] and hardly integrated into patient care. However, the use of naturopathic and complementary treatments among the population remains popular [5]. Andrew Weil, founder of the *Center for Integrative Medicine* at the University of Arizona points out the related problem that the integration of treatments “... [can develop] in a planned, thoughtful way, consistent with good science and ethics or it can develop haphazardly and recklessly. One can already see in the profusion of holistic and integrative clinics the lack of substance and planning that one would expect, given the fact that medical schools are not preparing physicians to navigate in this new world” [6].

From the point of view of the Committee for *Integrative Medicine and Plurality of Perspectives of the Society for Medical Education* (GMA), patient-oriented and team-based medicine, as required in the Masterplan 2020 for Medical Studies in Germany [7], cannot be implemented without naturopathic, complementary and integrative medicine being assigned a clear role within medical education. The committee feels responsible for developing concepts for a practicable, prudent and evidence-based implementation into medical studies. The students should be enabled to integrate corresponding treatment options and patient preferences more closely into decision-making and care processes in the future. This position paper was developed by the committee together with the teaching work group of the *Forum of University Work Groups on Naturopathy and Complementary Medicine* [https://uniforum-naturheilkunde.de/] in a multi-stage consensus process. The forum was set up in the 1990s as an association of scientists working at German-speaking universities in the field of naturopathy and complementary medicine and today has well over 100 members. The activities of the individual work groups of the forum aim to promote and publish scientific research, academic teaching and the clinical application of naturopathic and complementary medical diagnostic and treatment methods. The exchange between scientists within the forum is fostered using a cooperation network structure. Practical examples from teaching were collected via this network structure (see attachment 1 ), which show current options for implementing relevant content in medical education.

The aim of this position paper is to present the topic’s relevance for medical education, to clarify concepts, to reflect on the status quo in Germany in an international context and to present concepts for medical education. It ties in with international developments, especially in North America, as appropriate training concepts have already been developed and tested there. With this position paper, the committee would like to initiate an urgently needed discourse on the integration of naturopathy and complementary medicine into medical education, taking into account existing divergent paradigms [8], [9]. 

## 1. Introduction

The increase in lifestyle-related and chronic diseases [10] and the greater integration of patient wishes and preferences into health care is leading to a worldwide increase in preventive, complementary, non-pharmacological and self-initiated medical treatments [11], [12]. In Germany and Switzerland, for example, around 40% of the population make use of complementary treatment methods over the course of a year [5], and the proportion is significantly higher among oncology patients [13]. Complementary medical treatments that are not coordinated with conventional medical treatments harbor risks for patients [14]. On the other hand, complementary medical treatments coordinated with conventional treatments have the potential to positively influence the course of an illness and patient satisfaction [15]. Since there is little or no research and teaching in this subject area at many German medical faculties, future doctors are often unable to acquire the relevant knowledge and skills at university or only in isolated circumstance [16], [17]. As a result, many patients are not properly informed and cared for. For example, many chronically and mentally ill people turn to naturopaths, whose training is unregulated and their cooperation with doctors limited [18]. Oncology patients complain about a lack of information and a lack of treatment options with regard to naturopathic and complementary medicine [19]. A current study among breast cancer patients shows that only 16% of the physicians treating them integrated such treatments into the patient treatment and one in five women used such options without their knowledge [20]. Other studies also show that many patients use additional treatment options on their own without a doctor’s knowledge [19], [21]. This behavior is potentially dangerous and can, for example, lead to interactions and complications arising from the additional intake of health products during chemotherapy, thereby reducing the effectiveness of chemotherapy or increasing toxicity [22], [23]. Physicians should be trained to respond to patient needs and to embed appropriate treatment options into patient care.

### 1.1. Definition of terms

The way in which different treatment options are related to one another is subject to constant change and manifests itself in the use of different terms. With regard to teaching, it is necessary for this position paper to define various terms. From a historical perspective, the concept of alternative medicine was first established in Western culture, which attempted to counterbalance the increasingly scientific orientation and specialization of medicine into separate fields [24]. From the 1980s to the turn of the millennium, these parallel and complementary procedures and treatment continued to be developed. At the beginning of the 2000s, the term integrative medicine (IM) emerged, in which all treatment options are synergistically and cooperatively coordinated on the basis of scientific evidence for the benefit of the patient [4], [25]. 

The term **alternative medicine** is now only used by laypersons but no longer by official institutions and professional societies or in scientific publications. In the US, for example, the *National Center for Complementary and Alternative Medicine* (NCCAM) was renamed the *National Center for Complementary and Integrative Health* (NCCIH) in 2014. This demonstrates the overcoming of the dichotomous approach of alternative medicine [25]. The term **complementary medicine** (CM) was initially often used as a synonym of alternative medicine. In the official and scientific context its treatments are seen as complementary treatment options, which are used to prevent illness or to support conventional treatment [25], [26] and are applied by various professions.

In Germany there is also the term **naturopathy **(Naturheilverfahren). Naturopathy can be divided into **classic naturopathy** and **extended naturopathy **(erweiterte Naturheilverfahren). According to Sebastian Kneipp, classic naturopathy includes regulative therapy (including mind-body medicine), nutritional therapy, hydrotherapy, phytotherapy and exercise therapy [27]. Which procedures are part of extended naturopathy cannot be clearly determined. Some authors include methods such as neural therapy, traditional Chinese medicine (TCM), acupuncture, homeopathy and anthroposophic medicine, others define this term more narrowly [27], [28]. In contrast to the clearly-defined term of classic naturopathy, the terms (extended) naturopathy and complementary medicine are usually used as over-arching terms without there being much clarity regarding which treatments and procedures are included [29]. In the US health care system, mind-body medicine has established itself in medical care and is also gaining in importance in Germany. This includes behavioral medical approaches and techniques from the lifestyle elements exercise, relaxation, stress regulation and nutrition. It is based on the salutogenesis approach and aims to activate self-care and self-healing [30]. 

The 2013 WHO strategy contains an outlook for 2014 to 2023 and in addition uses the term **traditional medicine** and defines it as follows: “Traditional medicine has a long history. It is the sum total of the knowledge, skill, and practices based on the theories, beliefs, and experiences indigenous to different cultures, whether explicable or not, used in the maintenance of health as well as in the prevention, diagnosis, improvement or treatment of physical and mental illness” [2].

As part of this strategy, the WHO recommends that its 193 member states integrate traditional and complementary medicine (T&CM) into their health systems. The term combination T&CM preferred by the WHO is based on the differences that exist worldwide with regard to the available products and practices and their use [2]. 

Overall, in Western culture, the focus is shifting away from seeing medicine as a treatment intervention to a much more comprehensive picture of health care. Aspects such as personal responsibility, prevention, the environment, nutrition and social factors as well as the expertise of other medical professions are also taken into account. The term **integrative medicine** **(IM)** has established itself, especially since the the *Andrew Weil Center of Integrative Medicine* was set up at the University of Arizona in 1994 [4], [31]. It first appeared in Germany in 1992 in the journal *Therapeutikon* [32]. In recent years, various institutions and work groups have developed proposals for a definition of IM in which the concept of health is also central. Witt et. al. published a definition in 2017 that was developed in a consensual, international and interdisciplinary manner. It focuses on the aspect of well-being [33]. This position paper prefers the international definition of the *US Academic Consortium for Integrative Medicine & Health* for the term IM [34], which was expanded by Esch and Brinkhaus 2020 as follows: 

*“Integrative medicine and health reaffirms the importance of the doctor-patient relationship, targets the whole person, is informed by evidence, and employs all appropriate treatment, preventive, health-promoting, or lifestyle approaches, health care professionals and disciplines to achieve optimal health and healing; equally emphasizing the art and science of healing. It is based on a social and democratic as well as natural and healthy environment” *[35]*.*

Based on this definition, implications for teaching are derived below. Since individual naturopathic and complementary medical procedures in research and teaching must also be seen as isolated and not (yet) coordinated practices, the committee has agreed on the use of the term combinations **N**aturopathy, **C**omplementary and **I**ntegrative **M**edicine (N&CIM). In addition to the term integrative medicine, the term complementary medicine is thus also listed in parallel. The additional use of the term naturopathy is intended to establish a connection to the German-speaking tradition and nomenclature and the methods most frequently applied in Germany.

#### 1.2. Scientific evidence 

Evidence-based and scientific medicine requires that decisions should be made together with the patient as much as possible on the basis of proven effectiveness. According to the three pillars of evidence-based medicine, in addition to the values and wishes of the patient and the current state of clinical research, the individual clinical experiences of the doctor or therapist are also taken into account [4], [36]. 

In N&CIM there are now many studies on various procedures, some of which have a higher level of evidence and which have already found their way into health care practice. A comprehensive map of the current scientific evidence of numerous N&CIM procedures been compiled in the USA [37]. In Germany, N&CIM procedures are taken into account in a large number of the S3 guidelines, not least due to the admittance of the two scientific-medical specialist societies *Society for Phytotherapy e.V.* (SPT) and *Deutsche Gesellschaft für Naturheilkunde e.V. *(German Society for Naturopathy, DGNHK) in the umbrella organization of the *Association of the Scientific Medical Societies in Germany* (AWMF).For example, acupuncture, relaxation techniques, and massage can be used concomitantly to treat chemotherapy-induced nausea and vomiting in breast cancer patients [38]; mistletoe and mind-body medicine can contribute to improving the quality of life in patients with gastric cancer [39]. 

Gaining scientific knowledge and academic teaching go hand in hand, because university teaching aims to be methodologically sound and to justify its authority in the subject matter [40]. So far, German medical faculties lack full professorships for N&CIM. Endowed professorships and chairs are currently located at four state medical faculties in Berlin, Duisburg-Essen, Hamburg and Rostock. Two professorships have currently been made permanent at the Charité. Additionally, there are other chairs and endowed professorships, for example at the private University of Witten/Herdecke [41]. At these locations, it has already been possible to set thematic priorities in the area of N&CIM as part of the curriculum (see attachment 1 ). However, these chairs, which are predominantly funded by foundations, can hardly cover the cross-disciplinary and cross-methodological need for research in the field of N&CIM. With regard to research funds, the Hufeland Society, for example, has complained about the limited resources for conducting studies [42]. Against the background of high take-up, an international academic movement for IM and health promotion began to develop in the USA more than ten years ago. More than 70 university institutions joined to form the *Academic Consortium for Integrative Medicine and Health (ACIMH) *[43]. In view of the future challenges in the health care system, an annual budget is allocated for research in the USA, amounting to $152 million in 2020 alone. With these funds, concepts are to be developed as to how conventional medicine can be supplemented with evidence-based or evidence-informed complementary treatments [44]. So far there have been hardly any public research funds or efforts to systematically address N&CIM issues in the area of patient care and medical education in Germany. An exception is the research and practice initiative *Complementary and Integrative Health Care in Baden-Württemberg* (KiG BaWü), which has been funded by the Baden-Württemberg State Ministry for Social Affairs and Integration since June 2020. The work is carried out by the *Academic Center for Complementary and Integrative Medicine *(AZKIM) and the *Competence Network for Integrative Medicine *(KIM) and aims to optimally complement conventional medicine with naturopathic and other complementary medical treatment concepts and thus to provide patient care “hand in hand” in the future [45]. Additional appropriate initiatives could close existing gaps in quality and further develop integrative medicine, taking circumstances in Germany into account. This would also open up new perspectives for scientifically sound teaching on N&CIM oriented towards the future realities in care. It is necessary that teachers in the field of N&CIM have adequate conditions equal to those in other teaching and research fields in order to be able to implement research, teaching and patient care at the required academic level [46], [47].

#### 1.3. Naturopathy and CIM in German-speaking countries

In Germany, 60% of general practitioners state that they use N&CIM in outpatient clinical care and integrate procedures such as acupuncture, traditional Chinese medicine or phytotherapy [48], [49]. Irrespective of their specialization, doctors have the opportunity to acquire individual additional qualifications from the medical councils. Qualifications for N&CIM treatments can be acquired, for example, for acupuncture, homeopathy, manual medicine, naturopathy, physical medicine, balneology and medical climatology [50]. Around 70,000 doctors in Germany currently have an additional naturopathic qualification. Demand continues to be high [51].

In 1976, the new version of the Medicinal Products Act (AMG) for the first time listed phytotherapy, homeopathy and anthroposophy as “medicinal products of special treatment approaches” and included them in the catalog of services of the statutory health insurance schemes (Federal Law Gazette I, S 3394, of August 24, 1976, revised on December 12, 2005).

With the introduction of the curricular areas of naturopathy and homeopathy in the amendment of the Medical Licensure Act, complementary treatments were included in the training regulations for doctors back in 1988 (Federal Law Gazette I, S 1593, of July 14, 1987).

When the Medical Licensure Act was amended again in 2002, rehabilitation, physical therapy and naturopathy were firmly anchored in the medical studies curriculum as required and examination subjects in the cross-sectoral unit called QB 12, along with the option of including other complementary medical treatments as facultative subjects (Federal Law Gazette I, S 2405, 27 June 2002). In Chapter 16 (Therapeutic Principles) of the *National Competence-Based Catalog of Learning Objectives for Medicine 1.0* (NKLM) the following is stated [52]: “The graduates are able to describe and explain the therapeutic principles of physical medicine, naturopathy, complementary and alternative medical treatments, evaluate them critically and prescribe them appropriately if necessary.” However, the implementation is left to the individual medical faculties and is defined in the respective study regulations. A Germany-wide survey showed a very heterogeneous design of N&CIM content. In some places, this teaching content is completely omitted, for example due to a focus on the subject area of physical medicine and rehabilitation. In addition, the cross-sectoral unit QB 12 is only binding and mandatory for the standard degree courses in Germany and is not or only partially implemented in the model study courses [17]. Evaluation results show that the students welcome an in-depth teaching offer in the field of N&CIM [53], [54], [55]. Above all, students feel they need to be well trained so that they can better advise and, if necessary, treat patients in their future work [56]. 

The new Medical Licensure Act for medical studies, which is currently being restructured, brings with it the opportunity to develop standards and innovative formats for teaching N&CIM. Possible approaches to this have been formulated, for example, by the GMA* Integrative Medicine and Perspective Pluralism Committee*. These were published in appendix 8 of the statement by the advisory board and executive board of the GMA on the Masterplan for Medical Studies 2020. Here, among other things, there was an call for increased examination of different models of health and disease processing and for empowerment to integrate the patient's will into the care process in the sense of integrative medical care [57]. 

Through consistent education research, teaching in the area of N&CIM could be further developed and implemented into medical education in a targeted manner. To achieve this, current offers in teaching and training should be identified and a research strategy should be coordinated [58]. Last but not least, the combination of evidence-based teaching of N&CIM with practical experience could also promote critical thinking among students [55]. 

## 2. Competency development in the area of N&CIM

### 2.1. The status quo in medical education

In order to prepare students for a later patient-centered way of working, it is important that they learn how N&CIM can be appropriately integrated into treatment and therapy at the beginning of their training. In **North America**, the need to teach this content as part of medical degree courses was recognized early on. For example, in 2000, a work group for training at the *National Center for Complementary and Integrative Health* (NCCIH) drew up comprehensive recommendations to support medical faculties in the implementation of courses in CIM [59]. The NCCIH is a leading government agency for the scientific advancement of complementary and integrative health approaches [60].

Recent developments in the USA show a trend towards interprofessional education in CIM, which includes informal experiences, discussions and the exchange of ideas and resources of the individual professional groups based on social learning theories [34], [61]. Since 2000, the NCCIH has promoted a program called the *Complementary and Alternative Medicine Education Project*, which included 15 medical and nursing study programs. It emphasizes that nursing, even in traditional care models, must play a leading role in addressing the use of CIM and that corresponding courses must therefore be integrated longitudinally into the curricula [62], [63].

In 2012, the US *National Institute of Health* held the first international congress for CIM teachers in Washington DC. Interprofessional education was chosen as the central topic of the congress on the grounds that conceptual aspects such as efficient communication, patient-centered and relationship-oriented care play a central role here, just as with CIM [64]. 

In **Canada**, *Health Canada* initiated the *Complementary and Alternative Medicine in Undergraduate Medical Education* (CAM in UME) project in 2001 to prepare students for the integration of complementary treatment approaches into medical practice [65], [66]. The project was funded in 2006 with a budget of $77,750 to improve research into the status of CIM education in the health professions [67]. The goal was to enable students to discuss CIM with patients in an informed, non-judgmental manner. There should be no one-sided endorsement or rejection of individual techniques [68]. As part of this project, exemplary competencies for a CIM curriculum in basic training were also developed and presented. This includes the following areas: Basics (culture, evidence, placebo, usage and regulations), practices (chiropractic, massages, naturopathy) and patient care in the field of CIM (communication, patient information, CIM in chronic and oncological illnesses).

So far, there has been no public funding in **Germany** to develop corresponding courses in a comparable way. The* Integrative Medicine and Perspective Pluralism Committee* is committed to the development of a corresponding competency framework for university education in human medicine. In some cases, it was possible to incorporate individual aspects of this discourse into the development of NKLM 1.0, Chapter 16 (Therapeutic Methods) [52]. In order to sustainably anchor this in the graduate profile, more starting points are necessary. The Medical Licensure Act and the NKLM are currently being revised on the basis of the Masterplan adopted by the health and science departments of the federal and state governments in March 2017 [7], [57]. The aim is to adapt medical training to future challenges in health care and to more focus on skills and attitudes relating to the role of doctors, including scientific, communicative and interprofessional skills. Based on the measures of the Masterplan [69] highlighted by the Ministry of Education, possible starting points are named below:

1. According to measure 14 of the Masterplan, clinical and theoretical content should be linked from the first semester to the end of the training in order to create stronger and early practical relevance. This is suitable for the integration of N&CIM content. For example, communicative skills could be practiced using of real and standardized patients. With a focus on patient-centered care, it would be conceivable, for example, to develop case vignettes in which patients reject conventional treatment options or specifically ask about possible complementary options.
2. The greater weighting of scientific competencies is made clear by the required introduction of course certificate (measure 10 of the Masterplan). Scientific expertise and the integration of patient preferences into therapeutic decisions can be practiced well using N&CIM. Considering patient preferences is part of evidence-based action [36]. However, this also requires an informed decision with regard to possible side effects and interactions. Therapeutic options including N&CIM could be researched and checked by students with regard to their benefits and risk.
3. The strengthening of general medicine and the integration of teaching practices into medical training (measure 15 of the Masterplan) focuses on out-patient health care and better cross-sectoral integration. Especially in general medicine, students are often confronted with complex and chronic clinical pictures, which can be treated with supportive N&CIM. Patient preferences and the social environment play a major role here. During their studies, students should be prepared for this care reality and how to respond accordingly.
4. Measure 7 of the Masterplan provides for a mandatory offer of interprofessional lectures. The development of interprofessional and team-based teaching concepts involving all professions involved in the care process also includes the expertise of health professions in the area of N&CIM. Especially in the nursing, physiotherapy and midwifery professions, N&CIM are integrated into medical education and/or further education. For example, midwives can obtain further training in acupuncture, nursing professionals in the use of compresses and dressings, and physiotherapists in the use pf relaxation and movement training [70]. Here, for example, the joint creation of an interprofessional care plan in corresponding interprofessional courses could strengthen consideration of all treatment options [71].

#### 2.2. Development of a competence profile for medical education 

Based on the competencies of the ACIMH [34] and other literature [29], [47], [72], [73], [74], [75], the I*ntegrative Medicine and Perspective Pluralism Committee* developed a competency profile for medical education, consisting of seven competency areas. These range from practicing relational and patient-oriented medicine with a focus on one’s own personality to assuming responsibility in an increasingly globalized world. On the one hand, it is based on the knowledge that the attitude and persona of the doctor and their interaction with the patient has a decisive influence on the care process and treatment success [76]. On the other hand, the options and responsibilities of a doctor are seen as dependent on the political, economic, ecological and social systems [77], [78]. The individual competency areas with regard to N&CIM are presented in attachment 2 . 

Through the development of competencies, by placing the science and art of healing at the forefront of person- and patient-centered care, the foundations for optimal health maintenance and recuperation of every member of the population should already be laid down as part of medical education. According to the experts, a mandatory range of courses in which students acquire basic knowledge and skills for patient-centered care including N&CIM can have a positive long-term effect on the quality of patient care and, last but not least, on the development of medical faculties and teaching [79]. For example, the basic knowledge acquired during medical studies can be systematically referenced and expanded in medical further education.

In almost all N&CIM training programs described so far, students are expected to critically question newly acquired knowledge for potential use in patient care in dialog-oriented teaching formats [29], [54], [80], [81]. This can also be seen in the best practice examples listed in Chapter 3 and attachment 1 , which are implemented almost entirely as seminars with communicative and practical units. The integration of genuine case studies and practical exercises in teaching have proven successful to gradually familiarize students with the multitude of options in N&CIM [82] and can also be implemented in interprofessional seminars [71], [83]. They benefit from the integration of experts from different fields with clinical experience in the use of the respective procedures. One of the first publications on the teaching of CIM emphasizes that students develop a better understanding of the underlying treatment concepts and can better assess their practicability in patient care through hands-on experience of individual treatments [73]. Joos et al. also call for students to develop an authentic attitude towards naturopathy through self-awareness and reflection after completing the cross-sectoral unit QB 12 [29]. In order to learn how to see matters from the patient’s perspective and to practice appreciative communication, the involvement of real patients is considered to be particularly beneficial [84], [85]. Interprofessional practical seminars in the area of N&CIM can foster a common language, better understanding of patients and expansion of an individual, profession-specific perspective Interprofessional practical seminars in the area of N&CIM can foster a common language, better understanding of patients and expansion of an individual, profession-specific perspective [86]. Other interactive teaching methods, such as simulation-based learning [87] or online courses for the management of chronic diseases [88] have already proven effective in the interprofessional setting. The best practice examples compiled in this manuscript ) show further, diverse possibilities for integrating N&CIM into teaching. 

In order to consolidate medical core competencies, it is necessary in the long term not to label this topic as a special subject area in teaching. In concrete terms this could mean offering a separate cross-sectoral unit for this subject and not limiting it solely to QB 12. It is also possible to integrate the N&CIM content into other cross-sectoral units such as prevention and health promotion, or medicine of aging and the elderly, and in other specialist areas such as general practice, surgery, dermatology, gynecology, internal medicine, pediatrics or psychosomatics. With a longitudinal integration into existing clinical subjects, there is a risk that due to the high volume of teaching content, N&CIM content will be neglected or only taught superficially. The committee therefore initially prefers the implementation of the teaching of N&CIM in a specially designed compulsory module, to be offered as early as possible in medical degree courses, since students are more open to different treatment concepts at such an early stage [56], [89]. 

In the further course of studies, additional electives should offer students the possibility to set individual focal points. The competencies listed in attachment 1 represent the graduate profile. In which phases of a medical degree course these should be acquired must be tested and scientifically evaluated using corresponding pilot projects. N&CIM also offer a variety of options for integration into interprofessional teaching and general medicine. Initial studies indicate that an interprofessional patient history, for example, promotes a holistic view of the patient [90]. 

## 3. Best practice examples

Based on a survey among the members of the committee and the forum, best practice examples were recorded which have proven successful or were positively evaluated by the students. This compilation does not claim to be comprehensive. The relevant faculty teaching staff were asked to outline their range of courses in the area of N&CIM using specified key points. The results are summarized here and described in more detail in attachment 2 . 

The starting point for integration into medical studies at many medical faculties is the format of QB 12. In this regard, elective formats are offered in Freiburg, for example, in which the students learn about naturopathy in a practice-oriented, interactive and experiential manner. In Heidelberg, too, N&CIM content is taught as a compulsory and exam subject in the form of lectures and internships in small groups [29], [54]. In Tübingen, a series of lectures is offered as an introduction to the topic, the subjects of which can then be studied further in electives as part of the internship framework involving various professions and hospital visits. In addition to a two-semester lecture on two compulsory seminar days, the students in Duisburg-Essen gain an insight into the everyday life of a clinic for integrative medicine. During their elective course, they work on a ward for two weeks [91]. Internships and parts of the practical year (PY) are also offered at the Clinic for Integrative Medicine. 

In Rostock, interdisciplinary lectures have been held as part of QB 12 since 2003, and a naturopathy elective is also offered with a focus on phytotherapy and includes practical exercises. Since 2006, a second elective has enabled students to critically examine the relevant diagnostic and therapeutic devices. In addition, since 2004, students have been able to complete a 4-month rotation of their practical year in a rehabilitation clinic that has been designated as a teaching hospital for naturopathy. 

In the model study course introduced by the Charité in Berlin in the winter semester of 2010, the QB 12 offer is supplemented by interdisciplinary lectures and seminars with content from integrative medicine, such as practice-relevant concepts of IM, placebo and nocebo, multi-modal pain therapy, diet change, nutrition types, and a seminar on health and disease concepts. The format “Fundamentals of medical thinking and acting” in the 3^rd^ semester includes a clinical case discussion from the perspective of different complementary medical directions (e.g. naturopathy, osteopathy, mind-body medicine, Chinese medicine, Ayurveda) and shows basic structures and limits of these concepts using the example of a back pain patient. 

Interdisciplinarity and plurality of perspectives are the focus of the interactive case-based conference at Witten/Herdecke, where representatives from different medical directions (conventional medicine, homeopathy, traditional Chinese medicine, osteopathy and anthroposophic medicine) develop a treatment concept for a physically present patient and use this case to explain their approaches [92], [93]. Small groups develop the respective perspectives interactively and reflect on the specific contribution to integrative medicine and health care. 

In Heidelberg [94], [95] and Lübeck [96], [97], [98], medical students are taught together with students from other health professions in an interprofessional setting, for example with lecturers from different professions teaching in tandem. 

There are additional elective courses on offer, such as seminars on mindfulness-based stress management in Ulm. Here the participants learn how to develop serenity and inner qualities of consciousness through meditative exercises. 

In 2019, an endowed professorship for complementary medicine in oncology was established in the model study program in medicine in Hamburg-Eppendorf. During their medical studies, students receive brief overviews of N&CIM as part of their lectures. For interested students, there are additional opportunities in the second track preventive medicine unit, where CM is taught intensively for a week in the fifth semester. In addition, CM in oncology is also offered in the form of a seminar and traditional Chinese medicine in a student work group.

In the integrated complementary degree course in anthroposophic medicine in Witten/Herdecke, a longitudinal course has been developed, which takes up the topics and tasks of medical studies and expands and consolidates them through perspectives of anthroposophic medicine [99]. As part of the PY training station for IM, the students learn to apply integrative and anthroposophic medicine independently and under close supervision [100], [101], [102]. 

Practical relevance, interactivity, self-awareness, self-reflection and multi-perspectivity play an important role in many of these seminars. Fostering independent scientific judgment is also an essential learning objective. 

## 4. Discussion

Despite different priorities at each faculty, it was possible to find an agreement on the development of competencies and anchoring them in medical education on the basis of a common definition of terms. This position paper describes the current situation in Germany and, with a view to international developments, derives implications for the implementation of corresponding teaching offers into medical studies. From our point of view, there is a clear need for integrating the teaching of N&CIM competencies into medical education as obligatory content for all students in the long-term. The variety of best practice examples (see attachment 1 ) can serve as a starting point for the further development of teaching in the area of N&CIM, its accompanying research and cross-faculty exchange as well as initiating funding projects. However, it also shows a lack of evidence in relation to teaching, as only a few of the best practice examples presented were accompanied by scientific research. The committee sees itself as having a responsibility to promote appropriate research into teaching in this area. At the international level, it is clear that N&CIM are currently mainly implemented in interprofessional learning environments, although it must be noted that in most countries health care professionals are trained at universities and have more room decision-making [70]. In Germany, a restructuring and partial academization of the health care professions is currently underway [103]. The topic of interprofessionalism is now to be integrated into medical studies as obligatory and exam-relevant content for the first time [7]. Holistic care concepts are firmly anchored in the nursing and therapy professions and the corresponding professional groups make a significant contribution to health care in the area of N&CIM [104], [105]. From our point of view, the linking of N&CIM with interprofessionalism in teaching is forward-looking and should be taken up and developed further in future conceptual considerations. 

Overall, it is clear that teaching in the field of N&CIM has potential for developing a comprehensive graduate competency profile and can make a significant contribution to the acquisition and further development of medical roles and core competencies. Such a competency profile should be further developed in the future, taking into account all of the expertise, with a view to the entire area of medical and continuing education.

## 5. Outlook

In order to ensure the inter-connectivity of longitudinal competency development beyond medical education, it is important to keep an eye on specialist further education and to provide a basis during medical education on which further training can build. With the participation of the complementary medicine work group of the *German Association for General Medicine and Family Medicine* (DEGAM), a catalog of complementary medicine competencies for specialization in general practice was developed in a multi-stage, structured process. These build on the skills already acquired in medical degree courses (see above and NKLM) and are to be integrated into DEGAM’s competency-based curriculum for general medicine in the future [106]. This concept could also be transferred to other areas of specialization. 

Since the regulations in Switzerland differ significantly from those in Germany, both in terms of medical education and health care, the discourse in Switzerland will be presented in a separate position paper.

Another task is to design interprofessional learning opportunities in the area of N&CIM and to work out corresponding future fields of action and care scenarios. Overall, there is an urgent need for the systematic evaluation of relevant course offers in order to train future doctors in a structured and scientifically sound manner so they will be able to consider and integrate a wide range of care options in their future professional practice. 

## Acknowledgements

In addition to the authors involved in the manuscript, this position paper is supported by other leading representatives of naturopathy. Special mention should be made in particular of Holger Cramer, Gustav Dobos, Tobias Esch, Kristina Flägel, Doreen Jännichen, Christian Kessler, Karin Kraft, Jost Langhorst, Claudia Löffler, Andreas Michalsen, Georg Seifert and Diana Steinmann. 

## Note

The position paper was acepted by the GMA executive board at 31-01-2022.

## Competing interests

The authors declare that they have no competing interests. 

## Supplementary Material

List of naturopathy, complementary medicine and integrative medicine courses at medical schools in Germany

Competencies in medical education with special consideration of naturopathy, complementary and integrative medicine (N&CIM)
