# Supplementary material for: Naturopathy, complementary and integrative medicine in medical education – position paper by the GMA Committee Integrative Medicine and Perspective Pluralism
Source: GMS J Med Educ. 2022 Apr 14;39(2):Doc16. doi: 10.3205/zma001537 (PMC9174075; doi:10.3205/zma001537)
Supplement: List of naturopathy, complementary medicine and integrative medicine courses at medical schools in Germany [file JME-39-16-s-001.pdf]

Attachment 1: List of naturopathy, complementary medicine and integrative medicine courses at medical schools in Germany

Courses in the field of naturopathy (N), complementary medicine (CM) and integrative medicine (IM) at various faculties in Germany are presented below. These examples were compiled by the members of the *Integrative Medicine and Perspective Pluralism committee* and the *Forum of University Work Groups on Naturopathy and Complementary Medicine*. The list does not claim to be complete.

Content

1. **Cross-sectoral unit QB 12 and specialization opportunities offered as elective courses**
   1.1. Curricular courses on complementary and integrative medicine as part of the model study program in medicine, Berlin
   1.2. Courses in the field of complementary and integrative medicine, Duisburg-Essen
   1.3. Cross-sectoral unit QB 12, Freiburg
   1.4. Curricular courses, Rostock
   1.5. Courses on complementary and integrative medicine, Tübingen
   1.6. Elective Course: Mindfulness meditation course - stress management through mindfulness, Ulm
   1.7. Case-based conference as an example of a singular courses offering on plurality of perspectives, Witten/Herdecke

2. **Interprofessional courses**
   2.1. Interprofessional teaching module on complementary and integrative medicine (InterKIM), Heidelberg
   2.2. Interprofessional teaching module on complementary and integrative medicine, Lübeck

3. **Longitudinal Offers throughout Medical Studies**
   3.1. Courses in the field of naturopathy and complementary medicine as part of the model study program in medicine, Hamburg-Eppendorf
   3.2. The Integrated Curriculum for Anthroposophic Medicine (ICURAM), Witten/Herdecke

1. Cross-sectoral unit QB 12 and specialization opportunities offered as elective courses

1.1 Curricular courses on complementary and integrative medicine as part of the model study program in medicine, Berlin

Responsible parties: Project area complementary and integrative medicine, Institute for Social Medicine, Epidemiology and Health Economics, M. Ortiz, B. Brinkhaus Compulsory elective module: Clinic for Pediatrics with a focus on oncology and hematology (G. Seifert)
Execution:

- Naturopathy and integrative medicine (headed by B. Brinkhaus)
- Clinical Naturopathy (headed by A. Michalsen)
- Integrative and anthroposophic medicine (headed by H. Matthes)
- Clinic for Pediatrics with a focus on oncology and hematology (G. Seifert)

Curricular courses
Lecture: The healer is always right!? (2 teaching units of 45 minutes (TU), 9th semester)
Contents: On the basis of take-up data, the most frequently used treatments in complementary medicine are presented, taking into account their historical and philosophical background. A critical overview of the current state of research on efficacy and safety and the most important indications and limitations of these treatments is provided.
Main learning objective: The students should get to know the basics of the most important treatments in complementary medicine with their main indications and limits and be able to develop an open and critical attitude towards these treatments.

Seminar: Aspects of complementary medicine relevant to practicing naturopathy treatments/ complementary medicine (2 TU, 9th semester)
Contents: In this seminar, in particular practice-relevant learning content on complementary medicine is presented.
Main learning objective: The students should get to know and describe the practical use of various complementary medical treatments and be able to name seriousness criteria.

Basics of medical thinking and acting, case discussion of integrative and complementary medicine (32 TU, 3rd semester, elective)
Contents: Seminar plus practice on various CM treatment directions (naturopathy, traditional Chinese medicine, Ayurveda, hypnotherapy, mind-body medicine, osteopathy, anthroposophic medicine) with clinical reference to a back pain patient
Most important goal: In the seminar, the basic structures and the limits of complementary medical treatments are taught, critically reflected and compared with the basic structures of the scientific model of thought. The concept of integrative medicine or pluralism in medicine is developed based on a patient case report and critically discussed in the context of scientific evidence.

Compulsory elective module Integrative Medicine – Salutogenesis – Complementary Medicine. Clinical case seminar with patients from birth to old age (60 TU, 7th semester)
Contents: practical experience on site at the Immanuel Hospital Wannsee and Community Hospital Havelhöhe
Main learning objective: The students should acquire the ability to actively take different perspectives on patients in diagnosis and treatment, to create patho- and salutogenic multi-modal therapy concepts and to convey them to the patients. The focus is on the 3-pillar concept of evidence-based medicine with internal and external evidence and patient preference as the training objective of the course.

Characterization
Lecture and seminar on methods (classic naturopathy, phytotherapy, acupuncture and traditional Chinese medicine, manual medicine, relaxation methods, anthroposophic medicine, placebo effect)

Elective courses:
Practical implementation/application of methods (phytotherapy, collecting herbs, tea tasting, wraps and pads, acupuncture, meditation, shadowing)

Special didactic features
Elective courses: a lot of self-awareness in both elective courses

Response
Lecture & seminar: good evaluation
Elective courses: very good evaluation

Challenges
Lecture and seminar: late in the course of study (9th semester), restricted to limited content versus an overview of multiple treatments

Attachment 1 to Homberg A, Scheffer C, Brinkhaus B, Fröhlich U, Huber R, Joos S, Klose P, Kramer K, Ortiz M, Rostock M, Valentini J, Stock-Schröer B. *Naturopathy, complementary and integrative medicine in medical education. Positions paper by the GMA Committee: Integrative Medicine and Perspective Pluralism*. GMS J Med Educ. 2022;39(2):Doc16. DOI: 10.3205/zma001537

1.2 Courses in the field of Complementary and Integrative Medicine, Duisburg-Essen

Responsible parties: Chair of Naturopathy, Faculty of Medicine, University of Duisburg-Essen (Prof. Dr. G. Dobos)
Implementation of the courses: Prof. Dobos and colleagues

Courses
Cross-sectoral unit QB 12: Lecture series on naturopathy/CM (2 semesters)
Compulsory seminar: two full-day seminar days per student on site at the Clinic for Integrative Medicine
Elective Course: Mind-Body Medicine (14 days)

Goals/main learning objectives
Cross-sectoral unit QB 12: classify common methods in complementary medicine in terms of implementation and evidence and be able to explain their theory to the patient

Characterization
Cross-sectoral unit QB 12: *Lectures* on methods (classical naturopathy, phytotherapy, traditional Chinese medicine/acupuncture, physical, medicine, relaxation procedures, rehabilitation) for various indications
Elective Course: Participation in treatment groups as part of regulatory therapy. This allows the students to get to know mind-body medicine according to the Essen model in practice.
Seminar: practical implementation/application of traditional Chinese medicine methods, cupping, regulative therapy mind-body medicine

Special didactic features
Cross-sectoral unit QB 12: bedside teaching and self-application/exercise
Elective course: bio-psycho-social model of health and illness, treatment groups that promote motivation and self-reflection

Response
Cross-sectoral unit QB 12: very good evaluations (won the teaching prize for the best-evaluated course 3 times since 2003)
Elective course: 100% uptake

Challenges
To increase existing interest of the students, which by now is the case with the majority, in a relatively short period of time to such an extent that it can be called upon after graduation.

Literature
Cramer H, Haller H, Klose P, Langhorst J, Dobos G. Komplementärmedizin und Naturheilkunde von Anfang an – Das Interesse schon während des Studiums wecken. [Complementary and alternative medicine – piquing students' interest at the medical school]. Forsch Komplementmed. 2011;18:357-358. doi: 10.1159/000335327

1.3 Cross-sectoral unit QB 12, Freiburg

Responsible parties: Prof. Dr. Roman Huber
Execution: Employees of the University Center for Naturopathy and guest lecturers

Course description
Seminars on phytotherapy, naturopathy and physical medicine, lectures with patients and case studies on acupuncture/traditional Chinese medicine, anthroposophic medicine, homeopathy, naturopathy for cardiovascular diseases, gastrointestinal diseases, in oncology, for rheumatic diseases, in gynecology and in urology as well seminar on naturopathic research (a total of 30 TU).

Learning objectives (for reasons of space only for the seminars)
self-awareness and imparting practical knowledge of phytotherapy, naturopathy and physical therapy

Characterization (for reasons of space only for the seminars)
In a double hour each, the above schools of thought are presented in a practical and interactive way. In phytotherapy, typical substances classes (bitter substances, tannins, essential oils, etc.) of herbal medicinal products are presented according to indication (gastrointestinal, respiratory tract, calming and relaxing) and taught to the students in small groups (approx. 8 participants) using teas, tinctures and ready-made preparations they can experience.
In the naturopathy practice seminar, typical methods of naturopathy are discussed (liver compresses, manual diagnostics, cupping treatment) and their indications are presented in practice and carried out and experienced by the students themselves in small groups (approx. 8 participants).
The seminar on naturopathic consultation skills is about the art of consultation, including naturopathic aspects, patient preferences and self-management. In a double lesson, using patient actors the students practice conveying diagnosis and treatment concepts for functional diseases (irritable bowel syndrome) in small groups (4-6 participants).
In the practical seminar on physical treatments, important physical treatments (in particular hydrotherapy and balneotherapy, lymphatic drainage) are demonstrated in practice and carried out by the students themselves or among themselves.
The first three seminars mentioned are guided by doctors, the practical seminar on physical treatments by therapists.

Special didactic features
interactive in small groups, with a high proportion of self-awareness

Eligibility
Elective compulsory courses as part of cross-sectoral unit QB 12

Feedback/evaluation
good to very good

1.4 Curricular courses, Rostock

Responsible parties: Prof. Dr. med. Karin Kraft, endowed chair for naturopathy
Execution: Professorial staff and guest lecturers

Courses
Lectures: Cross-sectoral unit QB 12 (8th semester, summer semester only), also participation in lectures in cross-sectoral unit QB 12 and in the lecture series "Introduction to medical careers".
Electives: 1. Naturopathy (lecture with a focus on phytotherapy, small groups with patients).
2. Naturopathic devices in the doctor's surgery (seminar and internship)
Naturopathy elective course: In a rehabilitation clinic (naturopathy teaching hospital)

Learning objectives
Cross-sectoral unit QB 12: Classify common naturopathic/complementary medical methods in terms of implementation and evidence and be able to explain their theory to the patient.
Electives: Acquisition of basic knowledge (lecture) as well as self-awareness and teaching of practical knowledge, including how to deal with patients interested in naturopathy.
Elective course: Acquisition of basic knowledge and practical experience including self-awareness with natural healing methods that are regularly and optionally used in rehabilitation.

Characterization
Cross-sectoral unit QB 12: 2 semester hours per week, approx. one third teaching on naturopathy and complementary medicine. Since summer semester 2020, purely online course with access to instructional films, written face-to-face exam.
Electives with oral exam: Naturopathy: 2 semester hours per week + at least 6 hours of small group teaching with patients. Naturopathic devices in the doctor's surgery: 1 double hour seminar/week. Students can get to know the devices through self-experience.
Elective course: Integration into the processes of a rehabilitation clinic, patient care under supervision. Self-awareness in the application of naturopathic/physical treatments.

Special didactic features
Interactive in small group and seminar classes, high proportion of self-awareness.

Course credits
all courses

Feedback/evaluation
good/good

1.5 Courses on Complementary and Integrative Medicine, Tübingen

Responsible parties: Institute for General Medicine and Interprofessional Care: Prof. Dr. med. Stefanie Joos (Lead), Dr. med. Jan Valentini, Dr. med. Lioba Lohmüller
Execution: Professorial staff and guest lecturers

Courses
Cross-sectoral unit QB 12: Lecture series on naturopathy/complementary medicine
Elective course: applied complementary and integrative medicine
Note: Both courses take place in the same week, so that the elective course can be chosen as an additional in-deep course with practical relevance to cross-sectoral unit QB 12

Goals/main learning objectives
Cross-sectoral unit QB 12: classify common complementary medical methods in terms of distribution, evidence and safety and be able to explain their theory to the patient
Elective course: knowing and reflecting on the therapist and patient perspective of selected CIM procedures through practical self-experience and self-application

Characterization
Cross-sectoral unit QB 12: Lectures on methods (classical naturopathy, phytotherapy, acupuncture and traditional Chinese medicine, manual medicine, relaxation techniques, anthroposophic medicine, placebo effects) and indications (oncology and back pain), patient presentations.
Elective course: Practical implementation/application of methods (acupuncture, external applications, MBSR (Mindfulness-Based Stress Reduction), shadowing at the Filder Clinic)

Special didactic features
Cross-sectoral unit QB 12: Since summer semester 2020 as blended learning (voice over PowerPoint lecture and synchronous patient presentation)
Elective course: a lot of self-awareness, so students can apply what they have learned to themselves (e.g. relaxation techniques, MBSR)
Learning through emotion: Short films by a female patient: Due to the pandemic, the cross-sectoral unit QB 12 lecture was offered as an asynchronous course and the elective course as a synchronous online course from the summer semester 2020 onwards with very good response; newly introduced to the course were accompanying short films in which a female patient describes her experiences with various complementary medical methods in the course of her cancer.

Response
Cross-sectoral unit QB 12: good evaluation
Elective course: very good evaluation (in part rated as the best clinical elective course)

Challenges
Towards the end of the program (10th semester), many students would be interested in the elective after Cross-sectoral unit QB 12, but by then they do not have time or have already taken their elective course elsewhere.

Literature
Valentini J, Glassen K, Eicher C, Washington-Dorando P, Weinschenk S, Musselmann B, Steinhäuser J, Joos S. "Kritische Diskussion sollte mehr gefördert werden!"-Eine qualitative Auswertung der Lehrevaluation von Medizinstudierenden zur komplementärmedizinischen Lehre ["Critical discussion should be encouraged!" - a qualitative analysis of medical students' evaluation of a complementary medicine course]. Dtsch Med Wochenschr. 2018;143(14):125-30. doi: 10.1055/a-0575-6851

1.6 Elective course: Mindfulness meditation course – stress management through mindfulness, Ulm

Responsible parties: Prof. Dr. med Klaus Kramer (M.Sc.), Department of Integrative Medicine, Clinic for General and Visceral Surgery, University Hospital Ulm
Execution: Prof. Dr. med. Klaus Kramer (M.Sc.)

Learning objective

Opening of an inner path – (a training path of inner development) – based on the specific experience of each participant. Increasing the differentiation of one's own contents of consciousness such as thinking, feeling and wanting – internally and externally.

Characterization

Personal preliminary talks. Through practical instruction, participants are guided in practicing daily basic mindfulness exercises given in 8x2.5 hours (weekly) and 1x8 hours (mindfulness silence day, usually on the Saturday after the 6th day of the course): Body scan, walking meditation, standing yoga exercises, lying yoga exercises, sitting in stillness, breathing meditation, open awareness meditation, possibly Metta meditation.
Background on the theory, practice and science of meditation are taught; interactive practical exercises in the course. Structured course booklet with clearly formulated tasks for each week. Additionally accompanying, self-reviewed audio files for the exercises.

Didactic features

This course is about (self-) experience under practical guidance. The fact each path is individual is considered and addressed. The course was offered digitally for the first time in summer semester 2020 and as blended learning, completely digitally in winter semester 2020. The course meets the criteria of an MBSR course. The courses are accompanied scientifically (qualitative and quantitative evaluation).

Eligibility

Elective course certificate (28 semester hours, actual 42 semester hours), clinical and pre-clinical phases

Feedback/evaluation

The course is fully booked a few minutes after digital activation (there are currently three courses/semester). The teaching is mostly rated as extremely good, but it is noted that some participants do not find it easy engaging with their inner space – or with what is experienced in the process.

1.7 Case-based conference in integrative medicine as an example of a singular course offering on plurality of perspectives, Witten/Herdecke

Responsible parties: Diethard Tauschel, Christian Scheffer, Friedrich Edelhäuser, Integrated Curriculum for Anthroposophic Medicine (ICURAM) at the University of Witten/Herdecke

Learning objective
Reflecting on the procedure and treatment methods of different medical schools of thought based on a real patient encounter.

Characterization
In this all-day course for students in the clinical phase, the patient history of a real patient is taken by experts from four medical disciplines (e.g. conventional, homeopathic, anthroposophical and traditional Chinese medicine) in the seminar. Further findings as well as suspected diagnoses and treatment procedures are presented from the points of view of the different directions. Subsequently, the students deepen their knowledge of the medical system represented by the expert and then teach each other about what they have experienced in a "co-operative learning setting" in newly formed small groups. At the same time, the experts work together to develop an integrative medical treatment concept that is tailored to the patient. The results are then reported and discussed in the plenum.

Special didactic features
interactive, with a real patient, developed together with students, alternation between the experience of patient encounters and reflective elements

Eligibility
can be credited towards the cross-sectoral unit QB 12 certificate

Feedback/evaluation
The students rated their improved understanding of different medical directions and an integrative approach and medicine as very good.

Literature

Tauschel D, Scheffer C, Bovelet M, Brauer M, Escher M, Grosche O, Edelhäuser F. Case-based conference in integrative medicine: A pilot project to enhance learning and exchange of different medical concepts. EuJIM. 2009;1(4):197. doi: 10.1016/j.eujim.2009.08.104

Scheffer C, Edelhäuser F, Tauschel D, Hahn EG. Symposium Fallkonferenz Integrative Medizin: Vorbild für das Medizinstudium. GMS Z Med Ausbild 2007; 24 (1).

2. Interprofessional courses

2.1 Interprofessional teaching module on Complementary and Integrative Medicine (InterCIM), Heidelberg

Responsible parties: Dr. med. Katharina Glassen, Dr. Nadja Klafke
Department of General Medicine and Health Services Research, University Hospital Heidelberg

Learning objective
The aim is to encourage students to engage critically and scientifically with complementary medical treatment methods and to sensitize them to the individual care needs of patients in an interprofessional team.
The focus is on acquiring the following core competences:
1. Recognizing the need for care and respecting patient wishes.
2. Gain an overview of important CIM procedures and their evidence and recognize your own scope for action.
3. Knowing the competences of the different professions with regard to CIM and integrating them into the care plan.

Characterization
Since the summer semester 2018, the InterCIM teaching module has been offered jointly to students of medicine and the Interprofessional Health Care B.Sc.. Eleven courses each lasting 90 to 120 minutes are held on naturopathy and CM treatments. The selected topics are developed based on patient cases, taking into account the available evidence from the perspective of all participating professions. The respective application possibilities in patient care are discussed critically. It also raises awareness of the important link between basic research, fundamental research and patient care. Individual treatment techniques are tried out by the students themselves in practical exercises.

Special didactic features
The joint teaching approach is a special feature of the module. In order to teach the interprofessional perspectives in a practical way in the respective seminars, teachers from different professional groups are assigned jointly. In a cross-professional setting, the students work on an examination based on a self-conducted patient consultation in which the patient's perspective is sought and reflected on, taking into account the seminar content.

Eligibility
For students of the Interprofessional Health Care B.Sc. and medicine, the module is integrated as an elective and is credited accordingly.

Feedback/evaluation
The first evaluation results show a very positive assessment, both by the students and the lecturers. The students particularly appreciate the expertise of the lecturers as well as the opportunity to gain insights into many different treatment concepts and for professional exchange. The lecturers appreciate the small and interested group of students and the opportunity to contribute and discuss their knowledge of complementary medicine from a scientific perspective in a university setting.

Literature
Homberg, A., Klafke, N., Loukanova, S. und Glassen, K. (2019). Eine Brücke zwischen wissenschaftlicher Expertise und patientenzentrierter Versorgung schlagen: Ein interprofessionelles Wahlfachangebot Komplementäre und Integrative Medizin (InterKIM). Gemeinsame Jahrestagung der Gesellschaft für Medizinische Ausbildung (GMA), des Arbeitskreises zur Weiterentwicklung der Lehre in der Zahnmedizin (AKWLZ) und der Chirurgischen Arbeitsgemeinschaft der Lehre (CAL). Frankfurt am Main: Düsseldorf: German Medical Science GMS Publishing House. doi.org/10.3205/19gma033

Klafke, N., Homberg, A., Mahler, C., Loukanova, S. und Glassen, K. (2019). An interprofessional approach to teaching complementary healthcare delivery (CHD) to medical and healthcare students - evaluating the tandem method. 14th International Congress on Complementary Medicine Research (ICMMR). Brisbane: Advances in Integrative Medicine. doi.org/10.1016/j.aimed.2019.03.076

2.2 Interprofessional teaching module on Complementary and Integrative Medicine, Lübeck

Responsible parties: Dr. med. Kristina Flägel, Institute of General Practice, University Medical Center Schleswig-Holstein Campus Lübeck

Learning objectives

Students of medicine, nursing, physiotherapy, midwifery, speech therapy and occupational therapy can evaluate selected complementary medical procedures. Selected procedures are practiced on each other to gain self-awareness.

Overarching objective: to promote interprofessional exchange.

Characterization

Over the course of a semester, selected complementary medical procedures (phytotherapy, Chinese medicine, acupuncture, manual medicine/therapy, classical homeopathy, aromatherapy/care, neural therapy, anthroposophic medicine, nutritional therapy, osteopathy, mind-body medicine) are taught in a series of evening seminars using practical exercises with a case-centered focus. The content of the teaching module follows extensive preliminary work.

The seminars are supplemented by working through a case at home, six hours of shadowing followed by reflection in the form of an internship report and writing of a term paper which summarizes the evidence for a complementary medical procedure for a selected indication.

Special didactic features

The composition of the teaching staff represents a wide range of health professions and is as interprofessional as the student body. In a joint interprofessional case discussion, which is held on one of the seminar evenings, the lecturers bring together a wide variety of conventional and complementary medical approaches in an interprofessional exchange.

Course credits

For medical students, the teaching module must be completed as a pre-clinical or clinical elective course. As part of studies in nursing, physiotherapy, midwifery, speech therapy and occupational therapy, the teaching module is integrated into the curriculum as a compulsory elective module with 4 or 5 credit points.

Feedback/evaluation

So far, the teaching module has been taught three times (106 students, including 50 medical, 28 nursing and 28 physiotherapy students), each time with a waiting list in place due to the high level of interest from the students. The module was ranked 1.6 (on a scale from 1 to 6, with 1 being very good). The participants assessed their ability to advise patients and to discuss complementary medical procedures with colleagues as significantly better after completing the teaching module ($p = 0.002$ and $p = 0.001$, respectively). In the winter semester 2020/2021, 126 students were able to take the course online in a further round of teaching.

Literature

Prill P, Steinhäuser J, Flägel K. Determinanten interprofessioneller Zusammenarbeit – ein Mixed-Methods-Ansatz. Gemeinsame Jahrestagung der Gesellschaft für Medizinische Ausbildung (GMA), des Arbeitskreises zur Weiterentwicklung der Lehre in der Zahnmedizin (AKWLZ) und der Chirurgischen Arbeitsgemeinschaft Lehre (CAL), 25-28092019; Frankfurt am Main. Düsseldorf: German Medical Science GMS Publishing House; 2019.

Flägel K, Weber CL, Steinhäuser J, Götz K. Interprofessionelle Ausbildung in Komplementär- und Integrativer Medizin – Ergebnisse quantitativer Befragungen von Patienten und Studierenden zur Entwicklung eines interprofessionellen Lehrmoduls. Gemeinsame Jahrestagung der Gesellschaft für Medizinische Ausbildung (GMA), des Arbeitskreises zur Weiterentwicklung der Lehre in der

Zahnmedizin (AKWLZ) und der Chirurgischen Arbeitsgemeinschaft Lehre (CAL), 25-28092019; Frankfurt am Main. Düsseldorf: German Medical Science GMS Publishing House; 2019.

Flägel K, Prill P, Götz K, Weber CL, Steinhäuser J. Entwicklung eines interprofessionellen Lehrmoduls „Komplementäre und Integrative Medizin" – Implikationen für die Hausarztpraxis. 53 Kongress für Allgemeinmedizin und Familienmedizin 12-14092019; Erlangen. Düsseldorf: German Medical Science GMS Publishing House; 2019.

3. Longitudinal Offers throughout Medical Studies

3.1 Courses in the field of naturopathy and complementary medicine as part of the model study program in medicine, Hamburg-Eppendorf

Responsible parties: Prof. Dr. med. Matthias Rostock, endowed Professor for Complementary Medicine in Oncology
Execution: Employees and guest lecturers in addition to the endowed chair

Courses
Lecture: Introduction to complementary medicine and naturopathy (5th semester)
Lecture: Introduction to traditional Chinese medicine (8th semester)
Lecture: Complementary medicine in oncology (8th semester)
Seminar: Complementary medicine in oncology (8th semester)
Second track preventive medicine Complementary medicine and naturopathy (5th semester)

Learning objectives
The students can assess the importance of naturopathic and complementary medical treatment techniques for patients (e.g. with a tumor).
The students know the special aspects of naturopathic research and the status of scientific research on selected issues in complementary medicine.
The students can assess the possibilities and limits of complementary treatments for selected indications.
The students are able to inform themselves about their patients' questions about complementary medicine (e.g. with a tumor disease) so that they are able to give guiding advice.

Characterization
The lectures entail an overview of the various topics in naturopathy and complementary medicine for the students, intended as orientation. In the in-depth courses offered in the second track preventive medicine, individual aspects (for example phytotherapy, osteopathy, traditional Chinese medicine, homeopathy, nutritional medicine) are taught in more depth, mostly by established specialists in the respective areas, and the current state of scientific research and patient care is addressed. In the seminar on complementary medicine in oncology, essential questions in this specialist area are clarified using concrete patient cases and there is plenty of room for questions and discussions.

Response
good for lectures
very good for the seminar and second track with in-depth instruction.

3.2 Integrated Curriculum for Anthroposophic Medicine (ICURAM), Witten/Herdecke

Responsible parties: Diethard Tauschel, Christian Scheffer, Friedrich Edelhäuser, Integrated Curriculum for Anthroposophic Medicine

Learning objective
Enable graduates to provide integrative medicine and health care in which scientific medicine is supplemented by anthroposophic medicine.

Characterization: Example of a course offering throughout the entire length of medical studies
Over the entire duration of the medical degree course, ICURAM students can expand the content of the model study program towards the learning objective.
In years 1 and 2, seminars are offered on basic questions of anthropology to gain a deeper understanding of the organism and an increased perception.
In years 3 to 5, in integrative medical elective blocks and complementary seminars students learn how to take a multi-dimensional patient history and conduct a multi-dimensional clinical examination, how to cultivate an empathetic relationship and how a comprehensive treatment concept is developed on the basis of individual treatment needs.
The ICURAM training stations were developed for the Practical Year, in which the students treat patients independently under supervision using integrative medicine and thereby achieve a high level of patient orientation.

Special didactic features
The ICURAM was developed together with the students. Essential didactic elements are: Discovery l**e**arning, **s**tudent participation, **p**atient-based learning, **r**eflective learning, **i**ntegrated learning, **i**ntegrative approach, **t**eam-oriented learning (the ESPRI^{2}T Model).

Course credits
A number of requirements of the model study program can be completed in the ICURAM (clinical elective blocks, compulsory electives). Completion of the ICURAM also fulfills essential elements of the certificate for anthroposophic medicine of the Anthroposophic Medical Society in Germany.

Feedback/evaluation
55% of students take part in ICURAM seminars (16% fully, 18% partially, 22% occasionally), investing an average of 84hrs, 33hrs or 7.8hrs per year.

Literature
Scheffer C, Edelhäuser F, Tauschel D, Riechmann M, Tekian A. Can final year medical students significantly contribute to patient care? A pilot study about the perception of patients and clinical staff. Med Teach. 2010;32(7):552-7.

Scheffer C, Tauschel D, Neumann M, Lutz G, Valk-Draad M, Edelhäuser F. Active student participation may enhance patient centeredness: patients' assessments of the clinical education ward for integrative medicine. Evid Based Complement Alternat Med. 2013;2013:743832.

Scheffer C, Valk-Draad MP, Tauschel D, Büssing A, Humbroich K, Längler A, et al. Students with an autonomous role in hospital care - patients perceptions. Med Teach. 2018;40(9):944-52.

Scheffer C, Tauschel D, Neumann M, Lutz G, Cysarz D, Heusser P, et al. Integrative medical education: educational strategies and preliminary evaluation of the Integrated Curriculum for Anthroposophic Medicine (ICURAM). Patient Educ Couns. 2012;89(3):447-54
