# Supplementary material for: Naturopathy, complementary and integrative medicine in medical education – position paper by the GMA Committee Integrative Medicine and Perspective Pluralism
Source: GMS J Med Educ. 2022 Apr 14;39(2):Doc16. doi: 10.3205/zma001537 (PMC9174075; doi:10.3205/zma001537)
Supplement: Competencies in medical education with special consideration of naturopathy, complementary and integrative medicine (N&CIM) [file JME-39-16-s-002.pdf]

Attachment 2: Competencies in medical education with special consideration of naturopathy, complementary and integrative medicine (N&CIM)

<table>
<tr><td colspan="2">The graduates practice relational and patient-oriented medicine.
Central question: “How can future physicians be mindful, effective, and build a relationship with the patient that will positively impact health maintenance and healing?”</td></tr>
<tr><td>The graduates ...
➢ meet patients as equals, see them as equal partners and can build a trusting relationship.
➢ use or avoid certain words, statements, questions and facial expressions throughout the care process (patient history, diagnostics, treatment) in order to avoid negative reactions in patients and to initiate positive processes.
➢ know how to use their personality in ways that encourage and empower patients.
➢ recognize and consider the value of an ongoing doctor-patient relationship for patient-centered health care.</td><td>Notes: The attitude and person of the doctor and interaction with the patient has a decisive influence on the care process and treatment success. By practicing supportive communication and reflecting on their attitude and personality, students are supported in growing into a professional medical role, putting their own sensitivities aside and perceiving the needs of patients.</td></tr>
<tr><td colspan="2">The graduates perceive the patient in their individual totality.
Central questions: “With what needs and motivation does the patient come to see me? How is she/he affected as a whole person?”</td></tr>
<tr><td>The graduates ...
➢ have a basic anthropological understanding of the human being as a diverse, developing being whose autonomy and self-realization are impaired by illnesses.
➢ perceive the patient as a whole, including their social environment, recognize physical, vegetative and biographical dimensions of the illness and pay attention to functional connections.
➢ pick up on psychosocial aspects as well as existential questions when taking a patient’s medical history and in consultations and actively ask about the aims and motivation of the patients to influence health and healing through lifestyle as well as naturopathic and complementary medical treatment.
➢ take the patient's concerns and ideas about how to deal with the corresponding symptoms/illness seriously.</td><td>Notes: A prerequisite for a trusting relationship and the active involvement of patients in the care process is joint decision-making. The art of patient-centered care lies in recognizing suitable options in relation to the state of health and illness of individuals from the spectrum of one's own specialist knowledge and skills, while also taking into account the specific personality and their needs and using them advantageously. It is important to recognize, classify and adequately address the motivation for using N&CIM.</td></tr>
<tr><td colspan="2">The graduates justify treatment decisions through rational judgment.
Central question: “Which treatment techniques are suitable and what effects and risks can be expected with the respective techniques?"</td></tr>
<tr><td>The graduates ...
➢ know the principles of evidence-based medicine, which includes external evidence, internal evidence and the perspective and values of the patient.
➢ can research and assess the effects and side effects of conventional and unconventional methods on the basis of scientific literature.
➢ know the benefits and risks of frequently used N&CIM procedures and can form a balanced judgment based on scientific evidence.
➢ support individual decision-making and can make informed decisions together with the patient.
➢ reflect on the paradigms of different medical directions and can classify them in relation to overall health care.</td><td>Notes: The professional actions of doctors are based on decisions that are made based on the current state of scientific research and the advantages and disadvantages of diagnostic and therapeutic treatment options are weighed against each other. Under the premise of patient centering, this means that patients have the right to be informed comprehensively and knowledgeably about potential procedures. The goal is to support efforts on the part of patients while keeping safety in mind.</td></tr>
</table>

Attachment 2 to Homberg A, Scheffer C, Brinkhaus B, Fröhlich U, Huber R, Joos S, Klose P, Kramer K, Ortiz M, Rostock M, Valentini J, Stock-Schröer B. *Naturopathy, complementary and integrative medicine in medical education. Positions paper by the GMA Committee: Integrative Medicine and Perspective Pluralism.* GMS J Med Educ. 2022;39(2):Doc16. DOI: 10.3205/zma001537

**Graduates incorporate appropriate N&CIM treatments and lifestyle changes into patient care that serve to promote good health.**<br>Central question: “How can this patient be individually supported or treated?”	
The graduates ...<br>➢ know which naturopathic and complementary medical procedures and techniques are often used and what the motivations for their use are.<br>➢ have an overview and basic knowledge of frequently used techniques for lifestyle changes and resource activation, such as exercise, relaxation, nutrition, stress management.<br>➢ understand the principles of stimulus-response treatment in the area of N&CIM and can judge which reaction is triggered by which stimuli.<br>➢ have an overview and basic knowledge of frequently used treatment methods from the field of N&CIM, such as classic naturopathic treatments, acupuncture, homeopathy, osteopathy, Ayurvedic and Chinese medicine, anthroposophic medicine.<br>➢ can work out and justify which diagnostic and treatment options are suitable for which patients and how these can be used using exemplary clinical pictures and constellations.	*Notes:* Patients can only be comprehensively informed and cared for if physicians have an overview of all available options, including those in the area of N&CIM. Comprehensive care takes into account the lifestyle, resources and life goals of patients and, in addition to physical and mental healing, also aims to maintain health, alleviate pain and/or maintain and improve quality of life.
**The graduates work effectively and respectfully in a multi-professional and multi-disciplinary team.**<br>Central question: “How can different specialist and professional groups work together in a patient-centered manner?”	
The graduates ...<br>➢ know the roles, competencies and tasks of the professional groups relevant to patient care, including those who work in the field of complementary medicine.<br>➢ perceive the perspectives of the other professional groups on the patient, can classify and understand these perspectives and integrate them into the cooperative approach in an appreciative and respectful manner.<br>➢ in seeking a diagnosis, look beyond subject-specific boundaries, can taking a patient’s medical history in a multi-disciplinary, interprofessional care team and create a multi-professional and multi-disciplinary treatment plan.	*Notes:* No professional group or discipline alone can provide comprehensive patient care. Cooperation across disciplines, the perception of the other health care professions and the appreciation of the work they do is an important basis for successful, comprehensive patient-centered care in the area of N&CIM. There are many overlaps with N&CIM, especially in the pharmacy, physiotherapy, nursing and midwifery professions. Successful cooperation is fundamental here.

**The graduates are committed to a health-promoting lifestyle.**<br>Central questions: “How can incentives be set and conditions changed that promote a healthy lifestyle? How can future doctors set a good example?”	
The graduates ...<br>➢ know the basic definitions of health, illness and healing and are aware that physical, bio-psychosocial and biographical aspects influence illness, health and healing.<br>➢ take into account the subjective understanding of health, illness, healing and values of the patient in terms of their life, religion and spirituality.<br>➢ identify appropriate measures and options that patients can apply and carry out themselves to strengthen their health.<br>➢ know important self-efficacy strategies and can use them to support the active coping of patients.<br>➢ reflect on their own way of life in relation to their own resilience, burdensome stress factors and resources.<br>➢ take responsibility for their own health and are aware that they influence the behavior of patients in their function as role models.	*Notes:* At present, medical training is characterized by a fundamental principle of pathogenesis and the associated principles of disease cause factors and disease processes. The increasing number of complex and lifestyle-related diseases requires the balanced integration of the salutogenetic basic understanding, which primarily deals with the question of health maintenance. Genuine support for patients is only possible if future doctors also reflect on their own health behavior.
**The graduates assume their responsibility in an increasingly globalized world.**<br>Central question: “What are the effects of global changes on health? What are the economic and ecological consequences of medical intervention?”	
The graduates ...<br>➢ recognize connections between human health and political, economic, ecological and social systems.<br>➢ can assess the effects of their actions in terms of ecological and economic consequences and act in a resource-saving and sustainable manner with a view to costs.<br>➢ perceive global changes, especially with regard to the environment, epidemiology and health policy, and reflect on them in relation to medical action.<br>➢ assume social responsibility in their medical role and are committed to stable health policy conditions.	*Notes:* In an increasingly globalized world, health and disease are influenced by numerous factors such as pandemics, climate change and pharmaceutical patents. Medical action depends on global conditions and in turn has a direct or indirect impact on them. This basic understanding should be conveyed in medical education so that doctors are able to act with foresight and responsibility. Comparable points of reference can also be found, for example, in the Planetary Health concept.
